# Potentiating aminoglycoside antibiotics to reduce their toxic side effects

**DOI:** 10.1371/journal.pone.0237948

**Published:** 2020-09-02

**Authors:** Christopher R. Rosenberg, Xin Fang, Kyle R. Allison

**Affiliations:** 1 Department of Systems Biology, Columbia University Irving Medical Center, New York, NY, United States of America; 2 Department of Biomedical Engineering, Emory University and Georgia Institute of Technology, Atlanta, GA, United States of America; 3 Division of Infectious Diseases, Emory University School of Medicine, Atlanta, GA, United States of America; 4 Emory Antibiotic Resistance Center, Atlanta, GA, United States of America; Institut Pasteur, FRANCE

## Abstract

The lack of new antibiotics necessitates the improvement of existing ones, many of which are limited by toxic side effects. Aminoglycosides, antibiotics with excellent activity and low bacterial resistance, are hampered by dose-dependent toxic effects in patients (nephrotoxicity, ototoxicity). High antibiotic concentrations are often required to treat dormant, non-dividing bacteria, though previous studies show that aminoglycosides can be activated against such bacteria by specific metabolites. Here, we employed this mechanism to greatly boost the activity of low concentrations of aminoglycosides against prevalent Gram-negative pathogens (*Escherichia coli*, *Salmonella enterica*, and *Klebsiella pneumoniae*), suggesting that less toxic drug concentrations might be used effectively in patients. We go on to show that this effect improved treatment of biofilms, did not increase aminoglycoside resistance, and was due to the generation of proton-motive force (PMF). By single-cell microscopy, we demonstrate that stationary-phase cells, while non-dividing, actively maintain a growth-arrested state that is not reversed by metabolite addition. Surprisingly, within starved populations, we observed rare cells (3%) that divided without added nutrients. Additionally, we discovered that mannitol could directly protect human kidney cells from aminoglycoside cytotoxicity, independent of the metabolite’s effect on bacteria. This work forwards a mechanism-based strategy to improve existing antibiotics by mitigating their toxic side effects.

## Introduction

Over the past fifty years, antibiotic resistance has become a major public health hazard [1,2], causing tens of thousands of deaths [3] and costing billions of dollars to treat [3,4]. Creating new antibiotics is both costly and timely, evidenced by the dearth of FDA-approved antibiotics in recent years [5]. Thus, to combat the rise of antibiotic resistance, it is imperative to improve the antibiotics presently available.

Many antibiotics are limited by low potency or toxic side effects in humans. Given the important role of bacterial metabolism in antibiotic susceptibility [6–12], we reasoned that a strategy to rescue these antibiotics through metabolic intervention could improve problematic antibiotics and expand the current antibiotic arsenal. Aminoglycosides, one of the three major classes of antibiotics, are highly bactericidal and are commonly used to treat serious Gram-negative bacterial infections (*e*.*g*., *Escherichia coli*, *Klebsiella pneumoniae*, *Pseudomonas aeruginosa*) [4]. These drugs cause translational inhibition and misreading of tRNAs by targeting the 30S subunit of the bacterial ribosome [13] and have not succumbed to the same rise in resistance as fluoroquinolone, β-lactam, and cephalosporin antibiotics [14] (**Table 1**) [15–21]. For this reason, aminoglycoside use has increased in recent years [22]. However, clinicians must balance the efficacy of aminoglycosides with the potential for toxic side effects in patients, which include nephrotoxicity and ototoxicity [23]. As a result, aminoglycosides are typically reserved as a second-line antibiotic treatment. Adjuvants that lower the concentration of aminoglycoside necessary to kill bacteria could be transformative, and would particularly improve treatment of chronic and drug-resistant infections.

**Table 1:** Gram-negative pathogen resistance to antibiotics. Percent resistance (and reference) is indicated.

Species	Gentamicin	Tobramycin	Amikacin	Fluoroquinolone	Ampicillin	Cephalosporin
***E*. *coli***	11 [16]	9 [16],14 [17]	0.9 [17]	30 [15], 25 [17]	58 [17], 17 [18]	5.8 [19]
***S*. *enterica***	2.1 [80],33 [20]	-	0 [20]	11 [21]	70 [21]	-
***K*. *pneumoniae***	6.3 [16]	13 [16], 4.4 [17]	2.2 [17]	18 [15], 4.9 [17]	100 [17]	21 [19]

Percent resistance (and reference) is indicated.

**Fig 1:**
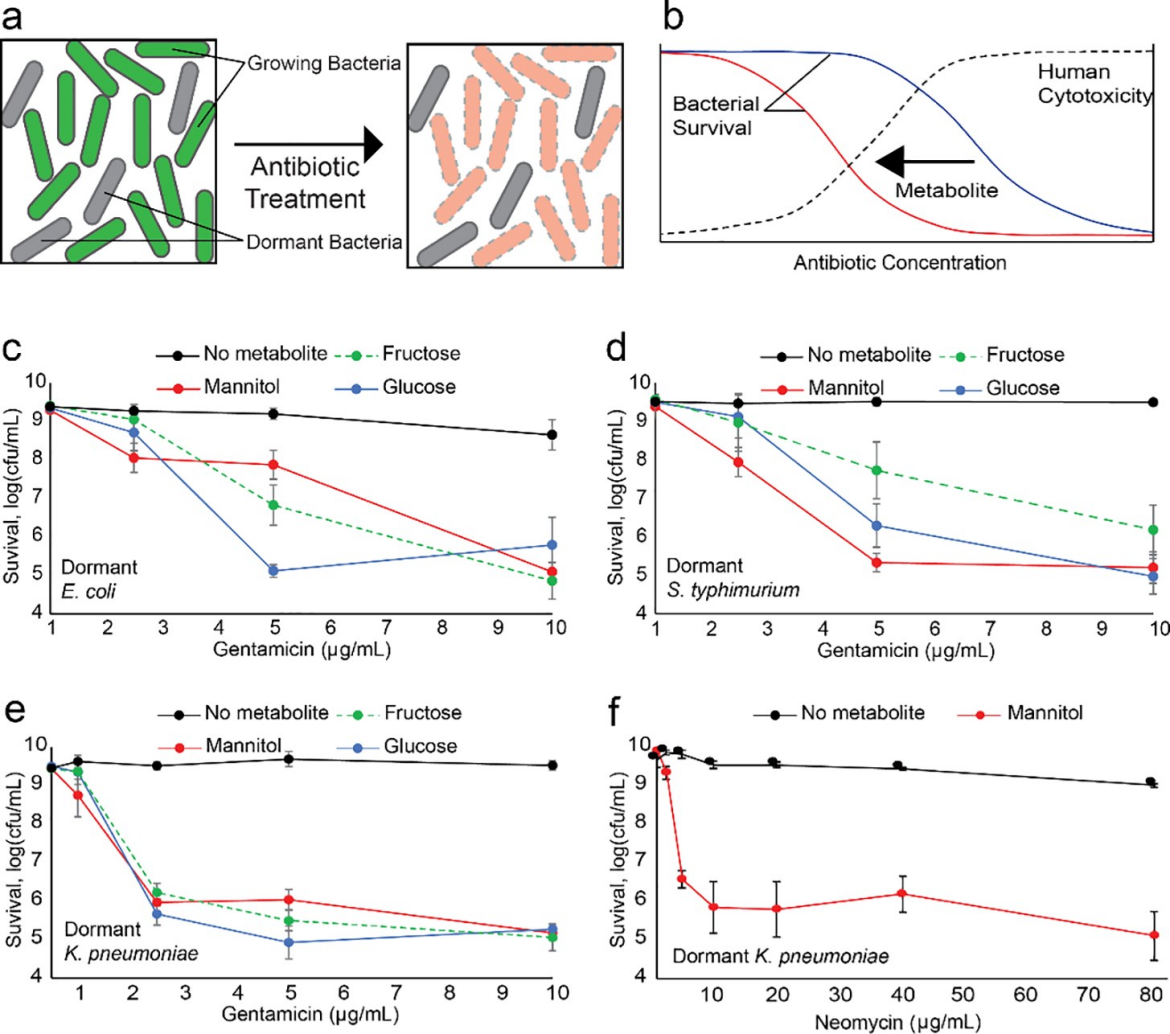
Metabolic potentiation to reduce effective aminoglycoside concentrations. **a**, Bacterial populations consist of a mixture of rapidly growing cells (green) and dormant, non-dividing cells (grey). Typical antibiotic treatment kills the majority of cells (orange) but has little impact on dormant, non-dividing cells, which necessitate higher antibiotic concentrations and longer treatment courses to eliminate the bacterial population. **b,** Aminoglycosides are dose-dependent antibiotics effective in treating many bacterial pathogens (blue line). However, their use carries the risk of toxic side effects as the higher antibiotic concentrations increase human cytotoxicity (dashed black line). With antibiotic-metabolite combinations (red line), we sought to reduce the concentration required to eliminate pathogens, thereby lowering the risk of human toxicity and “rescuing” aminoglycosides for broader use. **c,** Survival of stationary phase *E*. *coli* cells after 2-hour treatment with varying concentrations of gentamicin and no metabolite (black line), 10 mM fructose (green dashed line), 10 mM mannitol (red line), and 10 mM glucose (blue line). **d,** Survival of stationary phase *S*. *typhimurium* cells after 2-hour treatment with varying concentrations of gentamicin and metabolites. **e,** Survival of stationary phase *K*. *Pneumoniae* cells after 2-hour treatment with varying concentrations of gentamicin and metabolites. **f,** Survival of stationary phase *K*. *pneumoniae* cells following 2-hour treatment with varying concentrations of neomycin and mannitol.

Bacteria are capable of surviving antibiotic treatment without possessing genetic resistance factors. This phenomenon, called “phenotypic tolerance” or alternately “bacterial persistence” [24,25], plays an important role in the duration and outcome of treatments, as well as the total amount of antibiotic administered [26–29]. A primary mechanism of this phenotype results from a sub-population of cells entering a metabolically dormant state, allowing them to tolerate growth-dependent antibiotics [30–33]. Tolerance is a distinct phenotype from resistance which requires a genetic modification, allows cells to grow in the presence of antibiotics, and is measured by minimum inhibitor concentration. Meanwhile, metabolically active cells continue to grow, and remain susceptible to antibiotic treatment (**Fig 1A**). We previously uncovered a mechanism by which specific metabolites enabled high concentrations of aminoglycosides to eradicate *E*. *coli* persisters [6]. We reasoned that, by enhancing aminoglycosides, this mechanism could be used to lower the antibiotic concentration required to treat infections, and would thereby reduce the risk of nephrotoxicity and ototoxicity to patients (**Fig 1B**).

Aiming to demonstrate a strategy to mitigate aminoglycoside toxicity, we studied metabolic potentiation of multiple aminoglycosides over a range of concentrations against prevalent Gram-negative pathogens. We show that aminoglycoside-metabolite combination treatment significantly reduces the necessary antibiotic concentration to eliminate three major Gram-negative pathogens: *E*. *coli*, *Salmonella enterica* (serovar *typhimurium*), and *K*. *pneumoniae*. Moreover, we show this approach improves treatment of bacterial biofilms, and does not increase aminoglycoside resistance. Additionally, we provide evidence that proton-motive force (PMF) is both necessary and sufficient for this phenotype. Through tissue culture experiments in primary human kidney cells, we demonstrate that mannitol can attenuate aminoglycoside-induced cytotoxicity, suggesting an additional mechanism for minimizing nephrotoxicity independent of the metabolic effect on bacteria. Our results show that stimulating metabolism in a targeted manner can promote the activity of low doses of antibiotics. More generally, this strategy could improve antibiotics that lack potency or safety and, hence, could expand the current scope of existing antibiotics.

## Results

### Metabolites potentiate low gentamicin doses against Gram-negative pathogens

To test whether metabolic stimulation could reduce effective aminoglycoside concentrations, we supplemented aminoglycoside treatment with specific metabolites (glucose, mannitol, or fructose) in three prevalent Gram-negative pathogens: *E*. *coli*, *S*. *typhimurium*, and *K*. *pneumoniae* (see Materials and Methods). All cultures were grown to stationary phase, where bacteria enter a tolerant, non-dividing state after exhausting their nutrients, and were then treated for 2 hours, corresponding to the pharmacokinetic properties of aminoglycosides in patients [34].

We first tested gentamicin, the most commonly administered aminoglycoside [35]. Gentamicin alone demonstrated poor efficacy against *E*. *coli*, the primary cause of hospital-acquired Gram-negative infections [36], producing only 80% killing at the peak clinical concentration of 10 μg/mL (**Fig 1C**). Conversely, gentamicin-metabolite combinations produced 95% reduction even at a gentamicin concentration of 2.5 μg/mL, and approximately 4 orders of magnitude at peak concentrations. No killing was observed with 2.5 μg/mL gentamicin alone. Similar results were obtained for *S*. *typhimurium*, a pathogen closely related to *E*. *coli* and cause of gastrointestinal disease, urinary tract infections, and bacteremia [37]. Consistent with *E*. *coli*, all metabolites boosted elimination of *S*. *typhimurium* up to 4 orders of magnitude at peak doses, while gentamicin alone produced no killing (**Fig 1D**). As with *E*. *coli*, adding metabolite improved treatment by orders of magnitude, even at one-fourth the concentration of gentamicin.

This approach was most effective for *K*. *pneumoniae*, a pathogen more distantly related to *E*. *coli* and a major threat due to its development of resistance to many antibiotics and pernicious ability to spread in hospitals [38]. All three metabolites potentiated killing at gentamicin concentrations of 10 μg/mL by four and a half orders of magnitude (**Fig 1E**). Mannitol was the only metabolite that significantly enhanced killing at 1 μg/mL, producing 90% reduction. Treatment times extended to 8 hours nearly sterilized *E*. *coli*, but had no additional effect on *S*. *typhimurium* or *K*. *pneumoniae* (**S1a–S1g Fig in S1 File**). Moreover, we found this approach was also effective at killing bacterial persisters within the stationary phase population (**S2 Fig in S1 File**). Collectively, these data suggest that aminoglycoside-metabolite combinations are effective in treating Gram-negative bacteria at lower concentrations than the peak serum concentrations targeted by clinicians [39,40].

Having demonstrated potentiation using specific metabolites, we sought to determine whether their effects were due to catabolism, rather than an osmotic effect, by testing gentamicin combined with sorbitol (**S3a–S3c Fig in S1 File**), which resembled gentamicin treatment without metabolite. Though sorbitol can serve as a carbon source, starved bacteria preferentially consume glucose present in the inoculum before consuming sorbitol [41]. Additionally, we confirmed that metabolic potentiation occurs for the aminoglycoside class more broadly by testing two additional antibiotics, tobramycin and amikacin. In both cases, metabolites greatly improved killing by multiple orders of magnitude, except in the case of tobramycin in *S*. *typhimurium* (**S4a–S4f Fig in S1 File**). Only glucose potentiated tobramycin in *K*. *pneumoniae* and amikacin in *E*. *coli*. Metabolites did not potentiate aminoglycoside killing in rapidly growing cells, which are already highly susceptible to antibiotics, nor did they promote growth or antibiotic tolerance (**S5a–S5c Fig in S1 File**).

We considered this metabolite-potentiation might be applied to “rescue” neomycin, an aminoglycoside no longer used for intravenous therapy due to its severe toxic side effects [42]. We found that mannitol reduced the effective dose to treat *K*. *pneumoniae* below the 5 μg/mL serum threshold associated with human toxicity [43] (**Fig 1F**), while potentiating doses of 20 μg/mL or greater for both *E*. *coli* and *S*. *typhimurium* (**S6a–S6b Fig in S1 File**).

### Metabolites potentiate low aminoglycoside doses against bacterial biofilms

**Fig 2:**
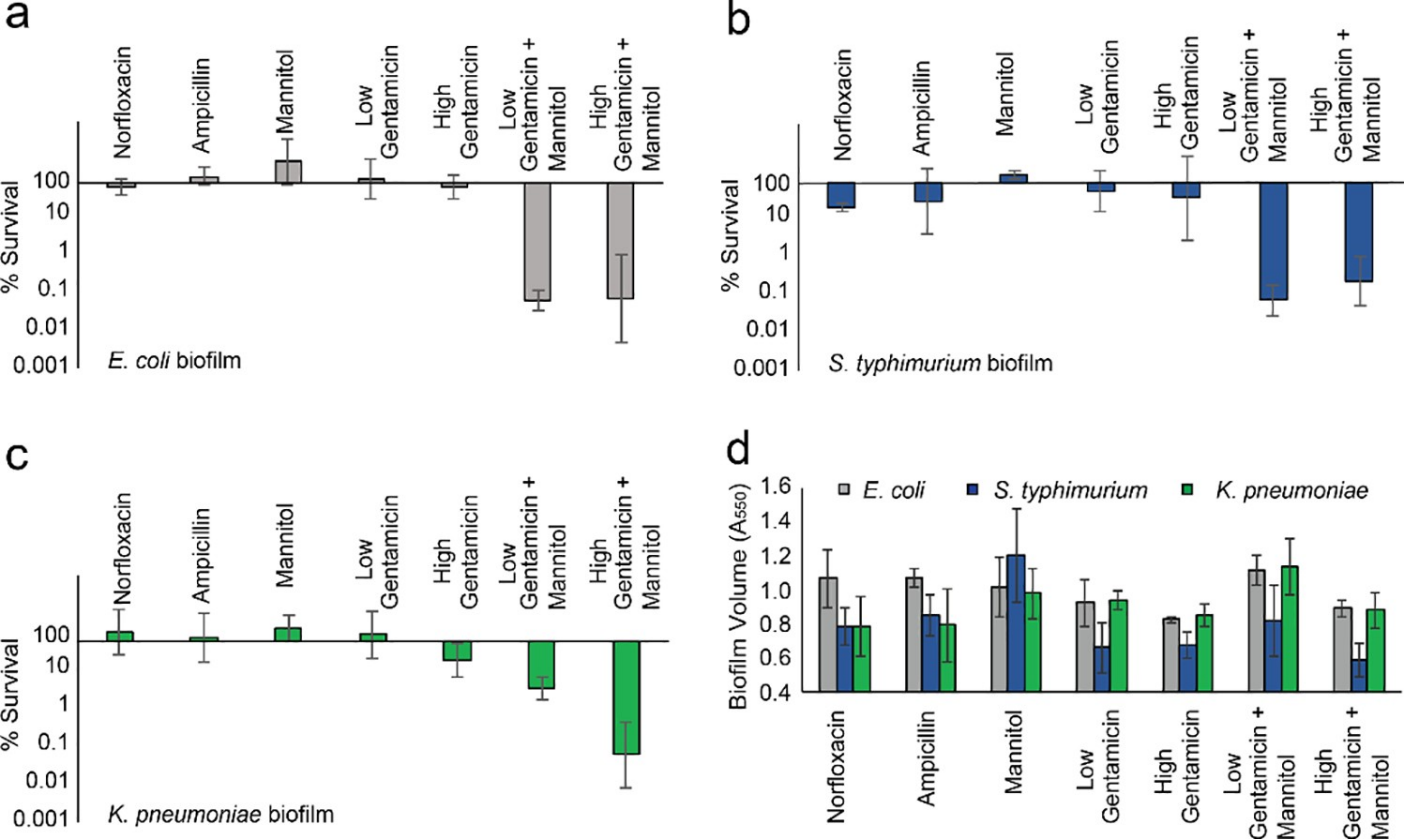
Metabolite-aminoglycoside combination improves treatment of Gram-negative biofilms. a, Survival of *E*. *coli* biofilms after treatment with norfloxacin (5 μg/mL), ampicillin (100 μg/mL), mannitol (10 mM), low gentamicin (1 μg/mL), high gentamicin (10 μg/mL), low gentamicin plus mannitol, and high gentamicin plus mannitol. b, Survival of *S*. *typhimurium* biofilms after treatment with norfloxacin, ampicillin, mannitol, low gentamicin, high gentamicin, low gentamicin plus mannitol, and high gentamicin plus mannitol. c, Survival of *K*. *pneumoniae* biofilms after treatment with norfloxacin, ampicillin, mannitol, low gentamicin, high gentamicin, low gentamicin plus mannitol, and high gentamicin plus mannitol. d, Crystal violet (CV) (0.1%) stain of *E*. *coli* biofilm (grey), *S*. *typhimurium* (blue), and *K*. *pneumoniae* (green) cells after treatment with norfloxacin, ampicillin, mannitol, low gentamicin, high gentamicin, low gentamicin plus mannitol, and high gentamicin plus mannitol. Absorbance values were recorded at 550 nm and were normalized by no-treatment CV stains for each strain. Plots represent mean +/- standard deviation for three or more replicates.

Bacteria commonly exist in structured communities known as biofilms, which can form on both abiotic and biotic surfaces, play important roles as reservoirs of infection, and exhibit high tolerance to antibiotics [32,44]. The tolerance of biofilms is a type of phenotypic resistance arising from the heterogeneity of cells [26,32,45], though, in some cases, tolerance results from poor penetrance of antimicrobials, particularly for β-lactam antibiotics [46]. Thus, we sought to determine the ability of metabolites to reduce the concentration of aminoglycosides required to eliminate biofilm cells. Biofilms of *E*. *coli*, *S*. *typhimurium*, and *K*. *pneumoniae* were grown for 48 hours and were then treated with antibiotics and mannitol. We found that gentamicin-mannitol treatments were highly effective at eradicating Gram-negative biofilm cells, whereas gentamicin alone had very little impact on biofilm cell viability. Specifically, for *E*. *coli* (**Fig 2A**) and *S*. *typhimurium* (**Fig 2B**), concentrations of gentamicin could be reduced ten-fold while still eliminating biofilm cells when mannitol was added. Low gentamicin plus mannitol treatment was less effective for *K*. *pneumoniae* (**Fig 2C**), achieving only 90% reduction compared to the 99% reduction with high gentamicin and mannitol. Ampicillin did not have any effect on the viability of any species tested, while norfloxacin only achieved killing in *S*. *typhimurium* by less than one order of magnitude.

We used crystal violet staining to determine if treatments were liberating cells from biofilms rather than killing them, a possible confounding factor with implications for treatment in patients. We found that low gentamicin treatments that included metabolite, despite potentiating killing, actually caused a mild increase in biofilm volume for *E*. *coli* and *K*. *pneumoniae*, although a small decrease for *S*. *typhimurium* was observed (**Fig 2D**). This suggests that combination treatments were in fact killing biofilm cells rather than dispersing them. Such a phenotype might be explained by the production of exopolysaccharide typically associated with aminoglycoside treatment [47]. Intermediate doses of gentamicin, when supplemented with mannitol, were similarly effective (**S7a–S7c Fig in S1 File**). To ensure cells were not detaching and growing planktonically, we tested the cell viability from the biofilm supernatants by flow cytometry and agar plating. For *E*. *coli* and *K*. *pneumoniae*, gentamicin-mannitol treatment resulted in a viability of 0.1% and 1%, respectively (**S8a–S8b Fig in S1 File**) (**Materials and Methods**). These findings suggest aminoglycoside-metabolite combinations may be useful for the treatment or prevention of infections resulting from Gram-negative biofilms.

### Metabolite supplementation does not increase aminoglycoside resistance

**Fig 3:**
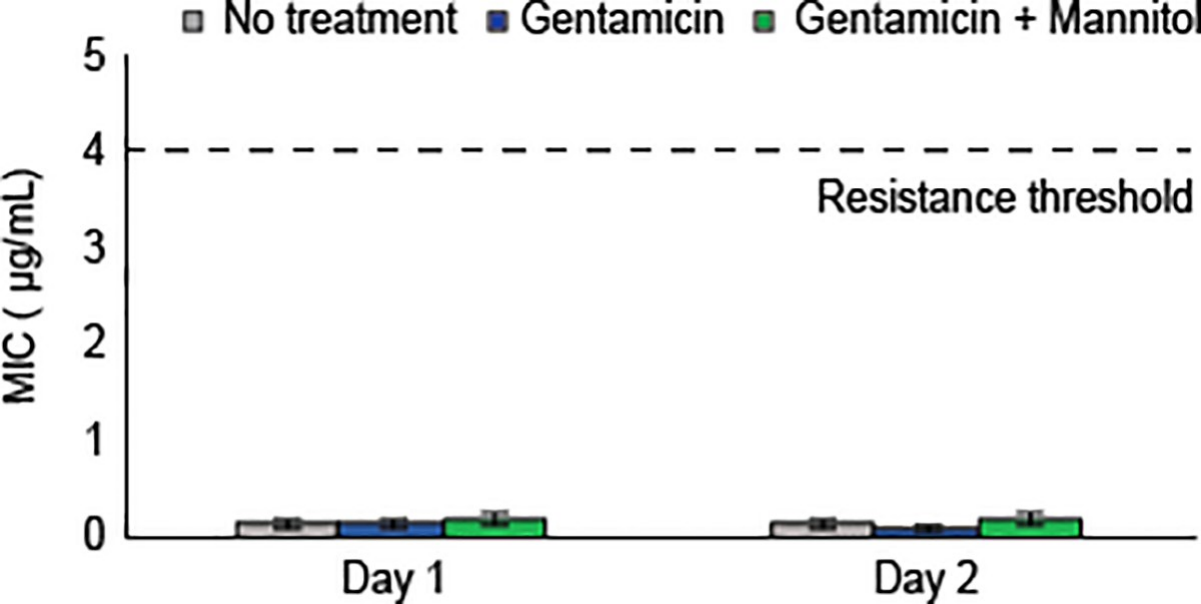
Metabolite potentiation does not accelerate development of aminoglycoside resistance. Minimum inhibitory concentrations following one- or two-day treatment of stationary phase *E*. *coli* with gentamicin (5 μg/mL) with or without mannitol (10 mM). Dashed line indicates minimum inhibitory concentration threshold for low-level gentamicin resistance in *E*. *coli*, as defined by the European Committee on Antimicrobial Susceptibility Testing [48]. Plots represent mean +/- standard deviation for three or more replicates.

Antibiotic dosing regimens can affect the development of antibiotic resistance. To study the possible emergence of aminoglycoside-resistant mutants, we investigated whether prolonged aminoglycoside-metabolite treatment might affect minimum inhibitory concentration (MIC), the standard measure of resistance. Stationary-phase cultures were treated on two consecutive days for two hours in the presence of gentamicin and mannitol, and MICs were determined (**Materials and Methods**). No significant difference was observed between untreated and gentamicin-mannitol cases, while a slight increase of 0.05 μg/mL occurred between gentamicin-mannitol and gentamicin-only treatments (**Fig 3**). Nonetheless, the MIC following gentamicin-mannitol treatment was twenty-fold lower than levels denoting genetic resistance [48].

### Role of metabolites in aminoglycoside potentiation

**Fig 4:**
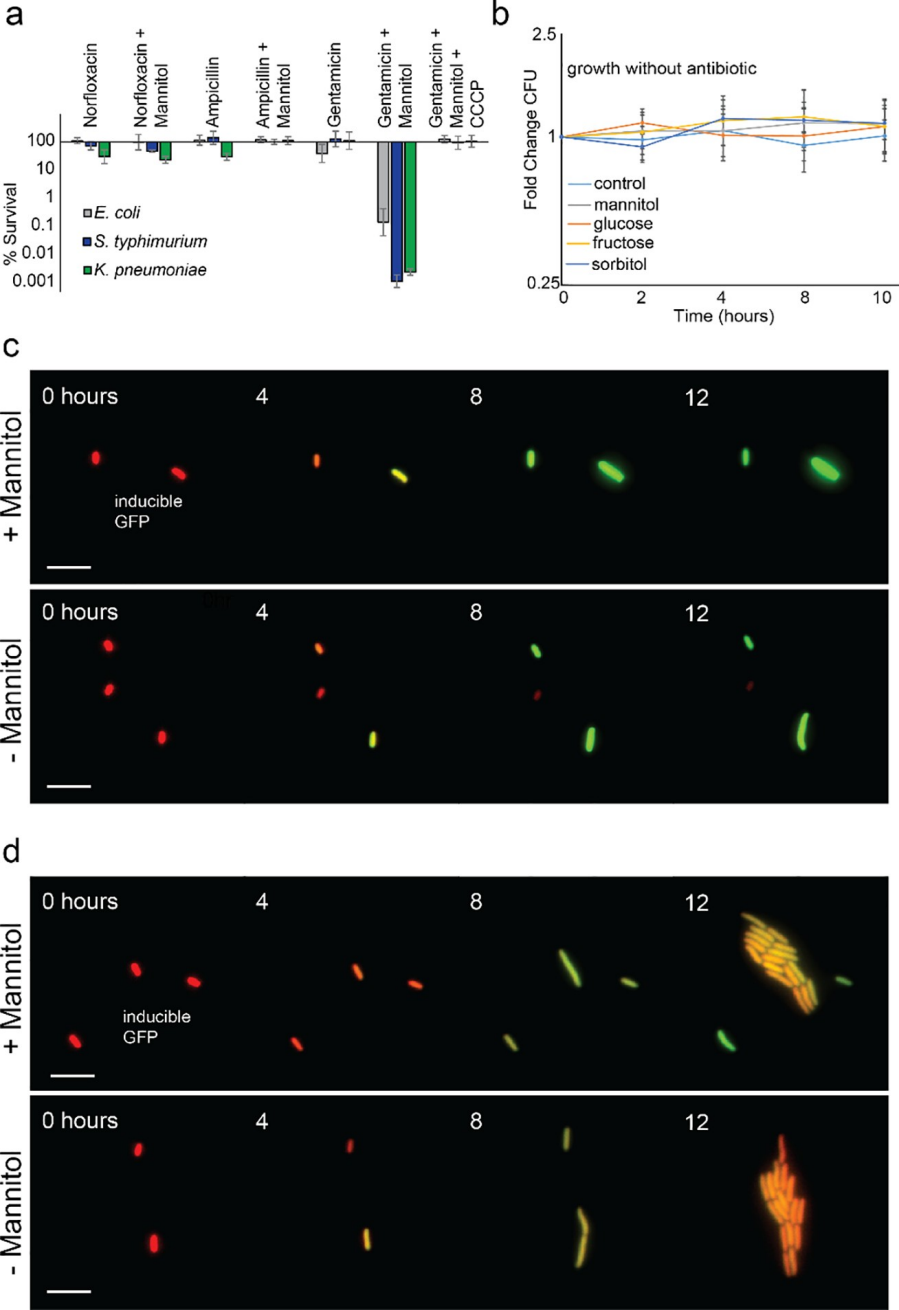
Metabolite-aminoglycoside killing is specific to aminoglycoside antibiotics, requires PMF, and does not require cell growth. **a**, Survival of stationary-phase *E*. *coli* (grey), *S*. *typhimurium* (blue), and *K*. *pneumoniae* (green) cells after treatment with gentamicin (10 μg/mL), gentamicin plus mannitol (10 mM), norfloxacin (5 μg/mL), norfloxacin plus mannitol, ampicillin (100 μg/mL), ampicillin plus mannitol, and gentamicin plus mannitol and CCCP (2.5 μg/mL). **b**, Growth of stationary-phase *E*. *coli* cultures after addition of 10 mM of the following metabolites: mannitol, glucose, fructose, and sorbitol. Plots represent mean +/- standard deviation for three or more replicates. **c**, Illustrative examples of stationary-phase *E*. *coli* cells (~400 observed) containing constitutively-expressed scarlet (red) and inducible GFP (green) tracked by microscopy at 37˚C after addition of 20 nM anhydrotetracycline and 10 mM mannitol (top) or no metabolite (bottom). Scale bar represents 6 μm. **d**, Illustrative examples of rare “cheater” cells (~3%) from the same experiments.

Past studies uncovered the importance of proton-motive force (PMF) in potentiating aminoglycoside against *E*. *coli* [6,49] and have indicated that metabolites do not return cells to active growth given that other classes of antibiotics are not potentiated [6,49]. We sought to test the specificity of potentiation in these Gram-negative species and treated each with norfloxacin or ampicillin in presence of metabolite (**Fig 4A**). Neither norfloxacin nor ampicillin were potentiated by mannitol in any species tested, suggesting that the processes of cell-wall synthesis and DNA replication targeted by these antibiotics remained inactive. Additionally, using carbonyl cyanide m-chlorophenyl hydrazone (CCCP), we showed that PMF was a requirement for potentiation in these species (**Fig 4A**), as was seen in *E*. *coli* persisters previously [6,49].

Curious of the inactivity of norfloxacin and ampicillin, we investigated if any of the metabolites used stimulated cellular growth over longer time scales. Similar to potentiation experiments, metabolites were added to stationary phase cultures, but no antibiotic was added and colony forming data was collected over an 8 hours rather than 2 hours (**Fig 4B**). We found that little-to-no growth was induced by any metabolite during this time frame, though all could serve as carbon sources if cultures were diluted in minimal media with extended growth periods. We reasoned that the bulk-scale nature of these experiment might mask more complicated dynamics occurring at the cellular level. To further explore the physiological effects of metabolite supplementation on stationary-phase cells, we performed single-cell imaging with an *E*. *coli* strain harboring inducible green fluorescent protein. Cells were grown to stationary phase without inducer then were added to agarose pads made from spent media (see Materials and Methods) with inducer in the presence or absence of mannitol. This experiment allowed us to simultaneously observe division and nascent protein synthesis resulting from metabolite supplementation. Dynamically tracking ~400 cells, we found that mannitol supplementation did not cause significant cell division (**Fig 4C**). Cells in both mannitol (+) and mannitol (-) conditions remained non-dividing for up to 12 hours, supporting the bulk-culture data (**Fig 4B**). Intriguingly, new protein synthesis was observed to a similar degree in both mannitol (+) and mannitol (-) samples. This suggests that, despite nutrient limitation, stationary-phase cells are capable of translation and adapting in response to their environment, adding to past findings in bacterial persisters [50]. Given that these cells tolerate aminoglycosides (which target translation) in the absence of mannitol, this result further suggests that the metabolite’s role in potentiation is to generate PMF, thereby facilitating antibiotic uptake.

Surprisingly, growth heterogeneity was observed in the late stages of these experiments: rare cells (~3%) in both mannitol (+) and mannitol (-) conditions began dividing around 8 hours and had formed microcolonies by 12 hours (**Fig 4D**). This is not due to heterogeneous availability of nutrients as dividing cells are present in them same environment as, and with little distance from, non-dividing cells. Instead, these cells appear to be phenotypic “cheaters” escaping from a regulatory blockade to utilize available nutrients. The behavior of these cells further indicates that the population majority is not arrested because of a lack of nutrients (also supported by the small difference between mannitol (+) and mannitol (-)), and instead may be stuck at a growth-phase checkpoint. In light of the observed translation (**Fig 4C**), these findings (**Fig 4D**) suggest that stationary-phase cells actively maintain a growth-arrested state.

### Mannitol protects kidney cells from aminoglycoside toxicity

Aminoglycoside nephrotoxicity, a primary limitation of aminoglycoside treatment, results from the accumulation of aminoglycosides in the kidneys, where they can induce apoptosis and necrosis by causing mitochondrial dysfunction and oxidative damage [51]. Mannitol is often used to protect against kidney toxicity during cancer chemotherapy with cisplatin, though the protective mechanism remains unclear. We hypothesized that mannitol, which is not a human metabolite and is commonly used clinically, might also protect against aminoglycoside-induced nephrotoxicity. To investigate this possibility, we cultured normal human primary renal epithelial cells (**Material and Methods**) and treated with gentamicin and mannitol, in combination and individually, for 72 hours before quantifying apoptosis and cell death by staining with Annexin-V FITC (indicating apoptosis) and propidium iodide (indicating necrosis). A gentamicin concentration of 2 mM (equivalent to 1033 μg/mL) was used, consistent with previous *in vitro* tissue culture studies in embryonic rat fibroblasts and porcine and canine renal cells [52]. The higher concentrations required for *in vitro* nephrotoxicity studies (~100 greater than ideal serum drug concentrations) may be due to aminoglycoside renal tubule concentrations reaching levels 10-times greater than serum concentrations [53].

**Fig 5:**
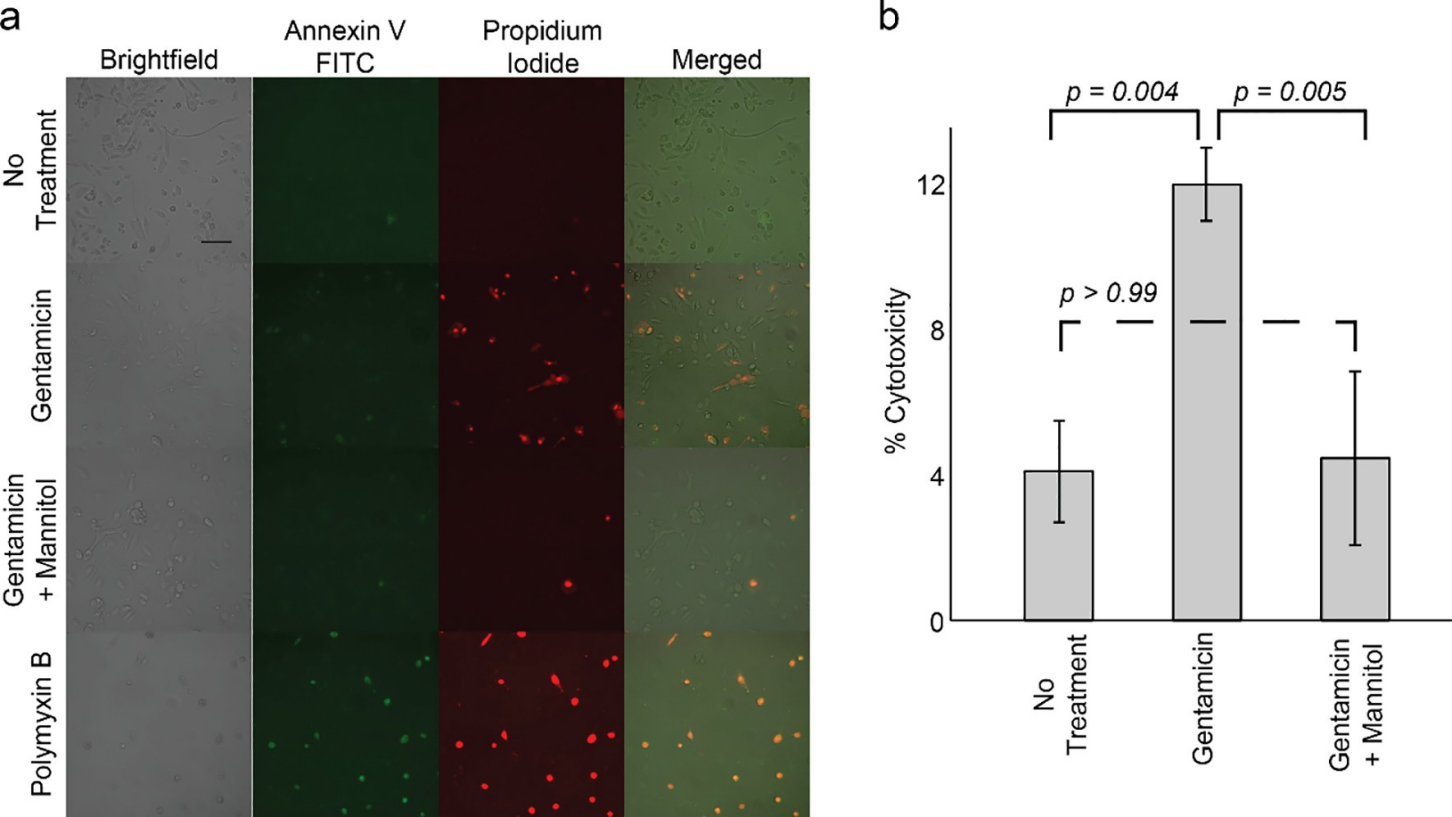
Mannitol reduces aminoglycoside-induced renal cytotoxicity. **a,** Bright field and fluorescent micrographs of normal primary human renal cells showing no treatment, gentamicin (2 mM or 1033 μg/mL), gentamicin plus mannitol (10 mM), or polymyxin B (0.5 mM). Annexin-V FITC (green) binds to phosphatidylserine, a lipid that presents extracellularly during cellular apoptosis, while propidium iodide (PI) (red) binds to DNA and indicates cell death. Images are representative examples. Scale bar represents 100 μm. **b,** Percent cytotoxicity (Annexin-V FITC or PI staining) of treatments. Plot represents mean +/- standard deviation for three biological replicates. Over 1,000 cells were quantified for each replicate.

We observed significant renal cytotoxicity in gentamicin-treated cells (12.0%) compared to untreated cells (4.1%) (**Fig 5B**), similar to previous studies [52]. However, adding mannitol greatly reduced cytotoxicity in gentamicin-treated cultures (4.5%). Annexin V and propidium iodide levels in gentamicin/mannitol samples were indistinguishable from untreated cells, suggesting that mannitol had blocked gentamicin toxicity. Polymyxin B, a macrolide antibiotic also used as a second-line treatment for serious Gram-negative infections, was used as a positive control given its severe nephrotoxicity (82.9%). The observed protective effect of mannitol was further evidenced by differences in cellular morphology (**Fig 5A**). The morphology of samples treated with gentamicin alone resembled samples treated with polymyxin B, as cells took on a spherical shape and appeared ready to detach from the plates. Alternatively, cells treated with gentamicin plus mannitol were morphologically indistinguishable from cells that did not receive treatment. These results indicate that mannitol may serve as a cytoprotectant against the kidney toxicity induced by aminoglycoside antibiotics. These findings reveal that specific metabolites can exploit bacterial metabolism while protecting human cells from antibiotic toxicity.

## Discussion

Gram-negative pathogens are a major medical problem with growing severity. There are now multidrug-resistant strains that cause infections with mortality rates higher than 50% in some cases [54]. In previous work, we rationally designed a strategy eliminate *E*. *coli* and *S*. *aureus* persisters with aminoglycosides. We have now shown this mechanism can reduce effective concentrations of multiple aminoglycosides against clinically-important Gram-negative pathogens (**Fig 1**). We further showed that it could improve treatment of biofilms (**Fig 2**) and did not contribute to increasing aminoglycoside resistance (**Fig 3**). Through investigating the physiological role of metabolites in potentiation, we uncovered evidence that stationary-phase cells, while non-dividing, have active protein synthesis and appear to maintain a dormant state that is not necessarily reversed by added nutrients (**Fig 4**). Additionally, we have demonstrated that mannitol, in addition to reducing the necessary aminoglycoside concentration to achieve high bacterial killing, may also protect against aminoglycoside cytotoxicity in human kidney cells (**Fig 5**). Aminoglycoside-metabolite combinations may allow clinicians broader and safer use of this major class of antibiotics.

Aminoglycosides are highly effective antibiotics and are resurgent as a consequence of alarming resistance rates to other antibiotics. Beyond their role in treating complicated infections, they are perhaps more integral to surgical practice, used in approximately 30% of prophylactic measures [55]. Aminoglycosides can cause nephro-[56] and ototoxicity [57] during treatment of infections, as well as during prophylaxis, despite relatively short-term drug exposure [58]. In an effort to mitigate aminoglycoside toxicity, clinicians have devised strategies to more effectively use aminoglycosides, including consolidated, high-dose therapies and selecting the least toxic aminoglycoside [59]. Although these strategies have shown promise, cytotoxic effects still occur, and patients who are experiencing decreased renal function, such as the elderly, are at particularly high risk. The approach proposed here could improve aminoglycoside efficacy while broadly reducing their toxicity, whether for treatment or prophylaxis, or administered intravenously or topically. The findings with neomycin suggest that it might be “rescued;” it is strongly antibacterial but deemed too toxic for intravenous use [42].

This strategy uses metabolites already administered clinically, at concentrations with proven safety [60]. Mannitol does not cause kidney toxicity below serum concentrations of 1000 mg/dl [61], a concentration (equivalent to 55 mM) over five times greater than used here. Metabolite potentiation of aminoglycosides might be applied in a variety of clinical scenarios. For example, inhaled mannitol is used in the management of cystic fibrosis to improve pulmonary function via improvement in airway surface hydration [62]. These patients also frequently have chronic pulmonary infections with Gram-negative species. Previous research showed that a combination of mannitol and tobramycin can improve killing of *Pseudomonas aeruginosa* by more than 5 orders of magnitude at a mannitol concentration of 10 mM [63], while a later study showed that tobramycin-mannitol combinations were ineffective in treating clinical isolates on CF-derived human airway cells grown in tissue culture [64]. However, this latter study only examined tobramycin, and at a concentration more than three orders of magnitude less than the mean peak airway concentration recorded in cystic fibrosis patients [65]. A greater range of concentrations may need to be tested in this system. Our results demonstrate that aminoglycosides do not perform uniformly as a class, and that different aminoglycosides and metabolites can have different activity depending on the bacterial species and treatment combination (**S4 Fig in S1 File**).

Mannitol can protect against chemotherapy-induced toxicity, such as cisplatin-nephrotoxicity or gentamicin-ototoxicity, by reactive oxygen species-scavenging and osmotic response [66,67]. The observed protection of kidney cells by mannitol (**Fig 5**) suggests that mannitol has potential as a standard adjuvant for aminoglycoside treatment in the future. As the concentrations used here were based on previous *in vitro* studies [6,52] future *in vivo* experiments will require pharmacologic optimization.

This strategy could seize on low rates of aminoglycoside resistance, significantly lower than rates of resistance for quinolones, β-lactams, or cephalosporin antibiotics (**Table 1**), and did not appear to increase resistance (**Fig 3**). Aminoglycoside cross-resistance is not a given, *i*.*e*. resistance to one aminoglycoside does not entail resistance to other aminoglycosides. For example, resistance to gentamicin rarely causes amikacin resistance [68] and *vice versa*. As our approach enhances a broad array of aminoglycosides, it could even prove effective against bacteria with a form of aminoglycoside resistance. Resultantly, this approach should provide a novel treatment for severe bacterial infections including those with multiple types of resistance.

In this work, we have demonstrated a strategy for treating Gram-negative pathogens and reducing aminoglycoside kidney toxicity by lowering effective antibiotic concentrations through metabolic potentiation. These findings suggest a way to broaden the use of existing antibiotics by limiting their toxic side effects, and add to growing efforts aimed at understanding and improving *existing* drugs as an alternative to discovering *new* ones [8,11,31,32,69–73].

## Materials and methods

### Bacterial strains and culture conditions

All experiments were performed with the following Gram-negative bacterial strains: *Escherichia coli* MG1665, *Klebsiella pneumoniae* subspecies *pneumoniae* (Schroeter) Trevisan, and *Salmonella enterica* serovar *typhimurium* LT2. *K*. *pneumoniae* (ATCC^®^ 43816^™^) and *S*. *typhimurium* (ATCC^®^ 700720^™^) were both purchased from the American Tissue Type Culture Collection. *Escherichia coli* MG1665Pro (F-, λ-, *Sp*^*R*^, *lacR*, *tetR*) with genomic-integrated PRO1-scarlet containing pEZ21-GFP was used in microscopy experiments.

All bacteria were cultured in 25 mL of Neidhardt supplemented MOPS defined medium (Teknova M2105, in which glucose is the primary carbon source) in 250 mL flasks at 37°C at 300 RPM. This media was specifically developed for the study of Gram-negative Enterobacteriaceae [74]. Cultures were grown for 16 hours or until OD_600_ 0.2–0.3, for stationary-phase (non-dividing) or exponential-phase (growing) experiments, respectively. For treatment, bacterial cultures were added in 1 mL volumes to 14 mL Falcon tubes already containing antibiotics and/or metabolites, then incubated at 37°C at 300 RPM for two hours. Two-hour treatment time was chosen to correspond to human pharmacokinetic characteristics of the antibiotics, representing the approximate half-life of aminoglycosides in normal patients [35], although some experiments were also performed using four- and eight-hour treatment times. Following treatment, cultures were serially diluted in phosphate-buffered solution (PBS) and plated on LB plates to determine colony-forming units.

### Antibiotics and chemicals

Metabolites were purchased from Fisher Scientific and Sigma-Aldrich. For all experiments, metabolites were diluted from concentrated stocks to 10 mM. Antibiotics and Carbonyl-cyanide m-chlorophenyl hydrazone (CCCP) were purchased from Sigma-Aldrich. Concentrations of gentamicin, tobramycin, and amikacin were consistent with target serum peak and trough concentrations used clinically [39,40,75,76].

### Microtiter plate biofilm assay

Biofilms were grown and quantified as previously described [77], with slight modifications. Briefly, overnight cultures of *E*. *coli*, *S*. *typhimurium*, and *K*. *pneumoniae* were diluted 1:100 into 100 μL of MOPS defined medium in 96-well plates (Corning) and incubated with gas permeable membranes (Breath Easy, RPI) at 37°C with no agitation for 48 hours. After washing twice with PBS, biofilms were treated with antibiotics and metabolites in fresh media for 2 hours at 37°C with gentle shaking at 150 RPM. After incubation, cells were washed twice with PBS. To quantify cellular viability, biofilm cells were liberated by sonication, serial diluted, and then plated on LB plates.

Biofilm production was quantified by crystal violet (CV) staining. After treatment, biofilms were washed twice with distilled water, incubated with 0.1% CV at room temperature for 15 minutes, and rinsed with distilled water four times to remove loosely associated bacteria. Plates were placed upside down for three hours to dry. Next, 30% acetic acid was added to each well to solubilize bound CV and plates were incubated for 15 minutes at room temperature. The solubilized CV were then transferred to a black-wall, flat-bottomed, 96-well plate, and absorbance was measured spectrophotometrically at 550 nm on a Biotech Synergy (Biotek).

To quantify the viability of cells in the biofilm supernatant, biofilms were grown as previously described, and then treated with gentamicin (5 μg/mL) or gentamicin and mannitol (10 mM) for 2 hours at 37°C with gentle shaking at 150 RPM. Next, 100 μL of supernatant was transferred into 0.5 mL 1X PBS and at least 500 cells were sorted by BD FACSAria II into 0.5 mL 1X PBS. Cells were spun down at 10 G for 2 minutes, then cells were resuspended in 50 μL of PBS and plated on LB agar plates. Cell viability was then calculated based on the number of colonies formed and theoretical number plated. These experiments were not performed for *S*. *typhimurium* after determining cells were made unviable by the flow cytometry apparatus, possibly as a result of the high-pressure fluidics or lasers that the machine uses to count cells.

### Acquisition of resistance via determination of minimum inhibitory concentrations

*E*. *coli* was treated with either gentamicin (5 ug/mL) or gentamicin plus mannitol (10 mM) for 2 hours as previously described. Cultures were then diluted 1:1000 and grown to OD_600_ 0.2–0.3 in 15 mL culture tubes, at which point cells were again diluted 1:1000 and grown overnight for 16 hours in 125 mL flasks. Cultures were then diluted 1:10,000 in the presence of a range of gentamicin concentrations in 96-well flat-bottomed plates and were incubated at 37°C for 18–20 hours, and MICs were determined corresponding to one-day treatment. Second-day treatments were then performed and subsequent MICs were measured in the same manner.

### Bacterial microscopy

Stationary phase cultures were diluted and added to 1% agarose pads made from spent medium prepared by filtering Neidhardt supplemented MOPS from stationary cultures. Images were obtained using a Leica DMi8 microscope equipped with a DIC HCPL APO 63X oil immersion objective, Hamamatsu ORCA-Flash 4.0 camera, and Lumencor Spectra-X light engine. Multi-channel, large-field, tiled-image time courses were collected using the Leica X software. Temperature was maintained at 37ᵒC throughout image acquisition using a stage-top incubator (Tokai-Hit). Excitation and emission for fluorescence microscopy was performed at 470 nm and 500–550 nm for green fluorescence and 510 nm and 592–668 nm for red fluorescence, respectively. Fluorescent exposures were 10 ms at 15% intensity and 20 ms at 20% intensity for green and red, respectively. Image scaling, processing, and cropping were performed uniformly across raw data using Leica Application Suite X (LAS X) software.

### Renal cell growth

Normal Human Renal Proximal Tubule Epithelial Cells (ATCC^®^PCS-400-010^™^) were cultured in renal growth medium (ATCC) with the supplements of fetal bovine serum (0.5%), triiodothyronine (10 nM), rh EGF (10 ng/mL), hydrocortisone hemisuccinate (100 ng/mL), rh insulin (5 mg/mL), epinephrine (1 mM), transferrin (5 mg/mL), and L-alanyl-L-glutamine (2.4 mM). Treatment media was supplemented with 0.001% Tween-20 to circumvent the established loss of aminoglycoside permeability typically observed in cultured human renal cells [78,79]. Cells were cultured at 37°C under 5% CO_2_ in monolayers in culture flasks or culture plates until 80% confluence before passage at a ratio of 1:3.

### Renal cell viability and cytotoxicity

Renal cytotoxicity was quantified with the Dead Cell Apoptosis Kit (ThermoFisher Scientific), which includes Annexin-V FITC and PI to quantify markers of apoptosis and cell death, respectively. Briefly, cells were grown in monolayers in 6-well plates as described above. After treatment with metabolite and antibiotic (at concentrations specified in **Fig 5**) for 72 hours, cells were stained in 1 mL PBS with Annexin V (5 μL), propidium iodide (1 μL), and Annexin-V binding buffer (360 μL) (see Dead Cell Apoptosis Kit) for 15 minutes in the dark. Stained cells were then fixed with 1% paraformaldehyde for 15 minutes on ice. Treatment with 0.5 mM Polymyxin B for 24 hours was used as a positive control. Cytotoxicity was determined by fluorescence microscopy on a Zeiss Apotome 2 (Carl Zeiss) based on Annexin V and PI staining. One hundred images were taken of each treatment condition per plate (totaling three wells per condition). Identical intensity and contrast scaling was applied across samples for fluorescent images. Annexin V- and PI-stained cells were counted for each condition and normalized by the number of cells to compute a percentage of cells experiencing cytotoxicity. Statistical analysis was performed using GraphPad Prism 8 software (GraphPad Software, Inc; La Jolla, CA). When comparing the means of three independent groups, a one-way analysis of variance (ANOVA) with Bonferroni’s multiple comparisons tests was performed.

### Renal cell microscopy

All images were collected using a Zeiss Apotome.2 microscope, using an EC Plan-Neofluar 10x/0.30 M27 objective and an Axiocam 503 camera. DIC, green fluorescent, and red fluorescent images were taken using exposure times of 2 ms, 750 ms, and 750 ms respectively. Excitation wavelengths for green fluorescence and red fluorescence were 495 nm and 592 nm, respectively, while emission wavelengths were 519 and 614, respectively. One hundred images were taken of each treatment condition per plate (totaling three wells per condition). Identical intensity and contrast scaling was applied across samples for fluorescent images. Annexin V- and propidium iodide-stained cells were counted for each condition and normalized by the number of cells to compute a percentage of apoptotic or necrotic cells.

## Supporting information

S1 File (PDF)

## References

[1] Davies J., and Davies D. (2010) Origins and evolution of antibiotic resistance. *Microbiology and molecular biology reviews*: *MMBR* 74, 417–433 10.1128/MMBR.00016-10 20805405 PMC2937522

[2] Ventola C. L. (2015) The antibiotic resistance crisis: part 1: causes and threats. *P & T*: *a peer-reviewed journal for formulary management* 40, 277–283 25859123 PMC4378521

[3] CDC. (2013) Antibiotic resistance threats in the United States, 2013.

[4] Fair R. J., and Tor Y. (2014) Antibiotics and bacterial resistance in the 21st century. *Perspectives in medicinal chemistry* 6, 25–64 10.4137/PMC.S14459 25232278 PMC4159373

[5] Boucher H. W., Talbot G. H., Bradley J. S., Edwards J. E., Gilbert D., Rice L. B., et al (2009) Bad bugs, no drugs: no ESKAPE! An update from the Infectious Diseases Society of America. *Clinical infectious diseases*: *an official publication of the* *Infectious Diseases Society of America* 48, 1–12 10.1086/595011 19035777

[6] Allison K. R., Brynildsen M. P., and Collins J. J. (2011) Metabolite-enabled eradication of bacterial persisters by aminoglycosides. *Nature* 473, 216–220 10.1038/nature10069 21562562 PMC3145328

[7] Conlon B. P., Rowe S. E., Gandt A. B., Nuxoll A. S., Donegan N. P., Zalis E. A., et al (2016) Persister formation in Staphylococcus aureus is associated with ATP depletion. *Nature microbiology* 1 10.1038/nmicrobiol.2016.51 PMC4932909 27572649

[8] Dwyer D. J., Belenky P. A., Yang J. H., MacDonald I. C., Martell J. D., Takahashi N., et al (2014) Antibiotics induce redox-related physiological alterations as part of their lethality. *Proceedings of the National Academy of Sciences of the United States of America* 111, E2100–2109 10.1073/pnas.1401876111 24803433 PMC4034191

[9] Kohanski M. A., Dwyer D. J., Hayete B., Lawrence C. A., and Collins J. J. (2007) A common mechanism of cellular death induced by bactericidal antibiotics. *Cell* 130, 797–810 10.1016/j.cell.2007.06.049 17803904

[10] Belenky P., Ye J. D., Porter C. B., Cohen N. R., Lobritz M. A., Ferrante T., et al (2015) Bactericidal Antibiotics Induce Toxic Metabolic Perturbations that Lead to Cellular Damage. *Cell reports* 13, 968–980 10.1016/j.celrep.2015.09.059 26565910 PMC4648786

[11] Lobritz M. A., Belenky P., Porter C. B., Gutierrez A., Yang J. H., Schwarz E. G., et al (2015) Antibiotic efficacy is linked to bacterial cellular respiration. *Proceedings of the National Academy of Sciences of the United States of America* 112, 8173–8180 10.1073/pnas.1509743112 26100898 PMC4500273

[12] Foti J. J., Devadoss B., Winkler J. A., Collins J. J., and Walker G. C. (2012) Oxidation of the guanine nucleotide pool underlies cell death by bactericidal antibiotics. *Science* 336, 315–319 10.1126/science.1219192 22517853 PMC3357493

[13] Davis B. D. (1987) Mechanism of bactericidal action of aminoglycosides. *Microbiological reviews* 51, 341–350 3312985 10.1128/mr.51.3.341-350.1987 PMC373115

[14] Gad G. F., Mohamed H. A., and Ashour H. M. (2011) Aminoglycoside resistance rates, phenotypes, and mechanisms of Gram-negative bacteria from infected patients in upper Egypt. *PloS one* 6, e17224 10.1371/journal.pone.0017224 21359143 PMC3040769

[15] CDDEP. Antibiotic Resistance.

[16] Rhomberg P. R., Deshpande L. M., Kirby J. T., and Jones R. N. (2007) Activity of meropenem as serine carbapenemases evolve in US Medical Centers: monitoring report from the MYSTIC Program (2006). *Diagnostic microbiology and infectious disease* 59, 425–432 10.1016/j.diagmicrobio.2007.05.009 17662557

[17] Lee S., Han S. W., Kim K. W., Song do Y., and Kwon K. T. (2014) Third-generation cephalosporin resistance of community-onset Escherichia coli and Klebsiella pneumoniae bacteremia in a secondary hospital. *The Korean journal of internal medicine* 29, 49–56 10.3904/kjim.2014.29.1.49 24574833 PMC3932395

[18] Tadesse D. A., Zhao S., Tong E., Ayers S., Singh A., Bartholomew M. J., et al (2012) Antimicrobial drug resistance in Escherichia coli from humans and food animals, United States, 1950–2002. *Emerging infectious diseases* 18, 741–749 10.3201/eid1805.111153 22515968 PMC3358085

[19] National Nosocomial Infections Surveillance, S. (2004) National Nosocomial Infections Surveillance (NNIS) System Report, data summary from January 1992 through June 2004, issued October 2004. *American journal of infection control* 32, 470–485 10.1016/S0196655304005425 15573054

[20] Olsen S. J., DeBess E. E., McGivern T. E., Marano N., Eby T., Mauvais S., et al (2001) A nosocomial outbreak of fluoroquinolone-resistant salmonella infection. *N Engl J Med* 344, 1572–1579 10.1056/NEJM200105243442102 11372008

[21] Chiappini E., Galli L., Pecile P., Vierucci A., and de Martino M. (2002) Results of a 5-year prospective surveillance study of antibiotic resistance among Salmonella enterica isolates and ceftriaxone therapy among children hospitalized for acute diarrhea. *Clinical therapeutics* 24, 1585–1594 10.1016/s0149-2918(02)80062-5 12462288

[22] Drusano G. L., Ambrose P. G., Bhavnani S. M., Bertino J. S., Nafziger A. N., and Louie A. (2007) Back to the future: using aminoglycosides again and how to dose them optimally. *Clinical infectious diseases*: *an official publication of the* *Infectious Diseases Society of America* 45, 753–760 10.1086/520991 17712761

[23] Huth M. E., Ricci A. J., and Cheng A. G. (2011) Mechanisms of aminoglycoside ototoxicity and targets of hair cell protection. *International journal of otolaryngology* 2011, 937861 10.1155/2011/937861 22121370 PMC3202092

[24] Levin B. R. (2004) Microbiology. Noninherited resistance to antibiotics. *Science* 305, 1578–1579 10.1126/science.1103077 15361616

[25] Balaban N. Q., Merrin J., Chait R., Kowalik L., and Leibler S. (2004) Bacterial persistence as a phenotypic switch. *Science* 305, 1622–1625 10.1126/science.1099390 15308767

[26] Allison K. R., Brynildsen M. P., and Collins J. J. (2011) Heterogeneous bacterial persisters and engineering approaches to eliminate them. *Curr Opin Microbiol* 14, 593–598 10.1016/j.mib.2011.09.002 21937262 PMC3196368

[27] Tuomanen E. (1986) Phenotypic tolerance: the search for beta-lactam antibiotics that kill nongrowing bacteria. *Reviews of infectious diseases* 8 Suppl 3, S279–291 3529321 10.1093/clinids/8.supplement_3.s279

[28] Fauvart M., De Groote V. N., and Michiels J. (2011) Role of persister cells in chronic infections: clinical relevance and perspectives on anti-persister therapies. *Journal of medical microbiology* 60, 699–709 10.1099/jmm.0.030932-0 21459912

[29] Vega N. M., Allison K. R., Khalil A. S., and Collins J. J. (2012) Signaling-mediated bacterial persister formation. *Nat Chem Biol* 8, 431–433 10.1038/nchembio.915 22426114 PMC3329571

[30] Rotem E., Loinger A., Ronin I., Levin-Reisman I., Gabay C., Shoresh N., et al (2010) Regulation of phenotypic variability by a threshold-based mechanism underlies bacterial persistence. *Proceedings of the National Academy of Sciences of the United States of America* 107, 12541–12546 10.1073/pnas.1004333107 20616060 PMC2906590

[31] Maisonneuve E., and Gerdes K. (2014) Molecular mechanisms underlying bacterial persisters. *Cell* 157, 539–548 10.1016/j.cell.2014.02.050 24766804

[32] Amato S. M., Orman M. A., and Brynildsen M. P. (2013) Metabolic control of persister formation in Escherichia coli. *Mol Cell* 50, 475–487 10.1016/j.molcel.2013.04.002 23665232

[33] Kussell E., and Leibler S. (2005) Phenotypic diversity, population growth, and information in fluctuating environments. *Science* 309, 2075–2078 10.1126/science.1114383 16123265

[34] Lortholary O., Tod M., Cohen Y., and Petitjean O. (1995) Aminoglycosides. *The Medical clinics of* *North America* 79, 761–787 10.1016/s0025-7125(16)30038-4 7791422

[35] Gonzalez L. S. 3rd, and Spencer J. P. (1998) Aminoglycosides: a practical review. *American family physician* 58, 1811–1820 9835856

[36] Gaynes R., Edwards J. R., and National Nosocomial Infections Surveillance, S. (2005) Overview of nosocomial infections caused by gram-negative bacilli. *Clinical infectious diseases*: *an official publication of the* *Infectious Diseases Society of America* 41, 848–854 10.1086/432803 16107985

[37] Cianflone N. F. (2008) Salmonellosis and the GI Tract: More than Just Peanut Butter. *Current Gastroenterology Reports* 10, 424–431 10.1007/s11894-008-0079-7 18627657 PMC2753534

[38] Munoz-Price L. S., Poirel L., Bonomo R. A., Schwaber M. J., Daikos G. L., Cormican M., et al (2013) Clinical epidemiology of the global expansion of Klebsiella pneumoniae carbapenemases. *The Lancet*. *Infectious diseases* 13, 785–796 10.1016/S1473-3099(13)70190-7 23969216 PMC4673667

[39] Muller A. E., Huttner B., and Huttner A. (2018) Therapeutic Drug Monitoring of Beta-Lactams and Other Antibiotics in the Intensive Care Unit: Which Agents, Which Patients and Which Infections? *Drugs* 78, 439–451 10.1007/s40265-018-0880-z 29476349

[40] Matzke G. R., Burkle W. S., and Lucarotti R. L. (1983) Gentamicin and tobramycin dosing guidelines: an evaluation. *Drug Intell Clin Pharm* 17, 425–432 10.1177/106002808301700604 6861633

[41] Lengeler J., and Lin E. C. (1972) Reversal of the mannitol-sorbitol diauxie in Escherichia coli. *J Bacteriol* 112, 840–848 10.1128/JB.112.2.840-848.1972 4563979 PMC251494

[42] Last P. M., and Sherlock S. (1960) Systemic absorption of orally administered neomycin in liver disease. *N Engl J Med* 262, 385–389 10.1056/NEJM196002252620803 14414396

[43] Grayson M. L. (2010) *Kucers' The Use of Antibiotic*, Hodder Education

[44] Levin B. R., Concepcion-Acevedo J., and Udekwu K. I. (2014) Persistence: a copacetic and parsimonious hypothesis for the existence of non-inherited resistance to antibiotics. *Current opinion in microbiology* 21, 18–21 10.1016/j.mib.2014.06.016 25090240 PMC4253300

[45] Lewis K. (2010) Persister cells. *Annual review of microbiology* 64, 357–372 10.1146/annurev.micro.112408.134306 20528688

[46] Anderl J. N., Franklin M. J., and Stewart P. S. (2000) Role of antibiotic penetration limitation in Klebsiella pneumoniae biofilm resistance to ampicillin and ciprofloxacin. *Antimicrob Agents Chemother* 44, 1818–1824 10.1128/aac.44.7.1818-1824.2000 10858336 PMC89967

[47] Hoffman L. R., D'Argenio D. A., MacCoss M. J., Zhang Z., Jones R. A., and Miller S. I. (2005) Aminoglycoside antibiotics induce bacterial biofilm formation. *Nature* 436, 1171–1175 10.1038/nature03912 16121184

[48] Jakobsen L., Sandvang D., Jensen V. F., Seyfarth A. M., Frimodt-Moller N., and Hammerum A. M. (2007) Gentamicin susceptibility in Escherichia coli related to the genetic background: problems with breakpoints. *Clinical microbiology and infection*: *the official publication of the* *European Society of Clinical Microbiology and Infectious Diseases* 13, 830–832 10.1111/j.1469-0691.2007.01751.x 17501975

[49] Meylan S., Porter C. B., Yang J. H., Belenky P., Gutierrez A., Lobritz M. A., et al (2017) Carbon Sources Tune Antibiotic Susceptibility in Pseudomonas aeruginosa via Tricarboxylic Acid Cycle Control. *Cell chemical biology*10.1016/j.chembiol.2016.12.015 PMC5426816 28111098

[50] Gefen O., Gabay C., Mumcuoglu M., Engel G., and Balaban N. Q. (2008) Single-cell protein induction dynamics reveals a period of vulnerability to antibiotics in persister bacteria. *Proc Natl Acad Sci U S A* 105, 6145–6149 10.1073/pnas.0711712105 18427112 PMC2329697

[51] Quiros Y., Vicente-Vicente L., Morales A. I., Lopez-Novoa J. M., and Lopez-Hernandez F. J. (2011) An integrative overview on the mechanisms underlying the renal tubular cytotoxicity of gentamicin. *Toxicological sciences*: *an official journal of the Society of Toxicology* 119, 245–256 20829429 10.1093/toxsci/kfq267

[52] El Mouedden M., Laurent G., Mingeot-Leclercq M. P., and Tulkens P. M. (2000) Gentamicin-induced apoptosis in renal cell lines and embryonic rat fibroblasts. *Toxicological sciences*: *an official journal of the Society of Toxicology* 56, 229–239 10869472 10.1093/toxsci/56.1.229

[53] Luft F. C., Patel V., Yum M. N., Patel B., and Kleit S. A. (1975) Experimental aminoglycoside nephrotoxicity. *J Lab Clin Med* 86, 213–220 168276

[54] Schwaber M. J., and Carmeli Y. (2007) Mortality and delay in effective therapy associated with extended-spectrum beta-lactamase production in Enterobacteriaceae bacteraemia: a systematic review and meta-analysis. *The Journal of antimicrobial chemotherapy* 60, 913–920 10.1093/jac/dkm318 17848376

[55] Khan A. K. A., P, V. M., Rashed M. R., and Banu G. (2013) A Study on the Usage Pattern of Antimicrobial Agents for the Prevention of Surgical Site Infections (SSIs) in a Tertiary Care Teaching Hospital. *Journal of clinical and diagnostic research*: *JCDR* 7, 671–674 10.7860/JCDR/2013/5323.2878 23730643 PMC3644441

[56] Mingeot-Leclercq M. P., and Tulkens P. M. (1999) Aminoglycosides: nephrotoxicity. *Antimicrob Agents Chemother* 43, 1003–1012 10.1128/AAC.43.5.1003 10223907 PMC89104

[57] Kalinec G. M., Webster P., Lim D. J., and Kalinec F. (2003) A cochlear cell line as an in vitro system for drug ototoxicity screening. *Audiology & neuro-otology* 8, 177–189 12811000 10.1159/000071059

[58] Wilson A. P., Sturridge M. F., and Treasure T. (1990) Aminoglycoside toxicity following antibiotic prophylaxis in cardiac surgery. *The Journal of antimicrobial chemotherapy* 26, 713–720 10.1093/jac/26.5.713 2079453

[59] Guo X., and Nzerue C. (2002) How to prevent, recognize, and treat drug-induced nephrotoxicity. *Cleveland Clinic journal of medicine* 69, 289–290, 293–284, 296–287 passim 10.3949/ccjm.69.4.289 11996200

[60] Hartwell R. C., and Sutton L. N. (1993) Mannitol, intracranial pressure, and vasogenic edema. *Neurosurgery* 32, 444–450; discussion 450 8455770

[61] Dorman H. R., Sondheimer J. H., and Cadnapaphornchai P. (1990) Mannitol-induced acute renal failure. *Medicine* 69, 153–159 10.1097/00005792-199005000-00003 2111870

[62] Bilton D., Bellon G., Charlton B., Cooper P., De Boeck K., Flume P. A., et al (2013) Pooled analysis of two large randomised phase III inhaled mannitol studies in cystic fibrosis. *J Cyst Fibros* 12, 367–376 10.1016/j.jcf.2012.11.002 23234802

[63] Barraud N., Buson A., Jarolimek W., and Rice S. A. (2013) Mannitol enhances antibiotic sensitivity of persister bacteria in Pseudomonas aeruginosa biofilms. *PloS one* 8, e84220 10.1371/journal.pone.0084220 24349568 PMC3862834

[64] Price K. E., Orazi G., Ruoff K. L., Hebert W. P., O'Toole G. A., and Mastoridis P. (2015) Mannitol Does Not Enhance Tobramycin Killing of Pseudomonas aeruginosa in a Cystic Fibrosis Model System of Biofilm Formation. *PloS one* 10, e0141192 10.1371/journal.pone.0141192 26506004 PMC4624634

[65] Geller D. E., Pitlick W. H., Nardella P. A., Tracewell W. G., and Ramsey B. W. (2002) Pharmacokinetics and bioavailability of aerosolized tobramycin in cystic fibrosis. *Chest* 122, 219–226 10.1378/chest.122.1.219 12114362

[66] Wood J. W., Bas E., Gupta C., Selman Y., Eshraghi A., Telischi F. F., et al (2014) Otoprotective properties of mannitol against gentamicin induced hair cell loss. *Otology & neurotology*: *official publication of the American Otological Society*, *American Neurotology Society [and] European Academy of Otology and Neurotology* 35, e187–194 10.1097/MAO.0000000000000342 24662629

[67] Pingle S. C., Mishra S., Marcuzzi A., Bhat S. G., Sekino Y., Rybak L. P., et al (2004) Osmotic diuretics induce adenosine A1 receptor expression and protect renal proximal tubular epithelial cells against cisplatin-mediated apoptosis. *The Journal of biological chemistry* 279, 43157–43167 10.1074/jbc.M405666200 15272017

[68] Kotra L. P., Haddad J., and Mobashery S. (2000) Aminoglycosides: perspectives on mechanisms of action and resistance and strategies to counter resistance. *Antimicrob Agents Chemother* 44, 3249–3256 10.1128/aac.44.12.3249-3256.2000 11083623 PMC90188

[69] Grant S. S., Kaufmann B. B., Chand N. S., Haseley N., and Hung D. T. (2012) Eradication of bacterial persisters with antibiotic-generated hydroxyl radicals. *Proceedings of the National Academy of Sciences of the United States of America* 109, 12147–12152 10.1073/pnas.1203735109 22778419 PMC3409745

[70] Morones-Ramirez J. R., Winkler J. A., Spina C. S., and Collins J. J. (2013) Silver enhances antibiotic activity against gram-negative bacteria. *Science translational medicine* 5, 190ra181 10.1126/scitranslmed.3006276 PMC3771099 23785037

[71] Palmer A. C., and Kishony R. (2014) Opposing effects of target overexpression reveal drug mechanisms. *Nature communications* 5, 4296 10.1038/ncomms5296 24980690 PMC4408919

[72] Zhou A., Kang T. M., Yuan J., Beppler C., Nguyen C., Mao Z., et al (2015) Synergistic interactions of vancomycin with different antibiotics against Escherichia coli: trimethoprim and nitrofurantoin display strong synergies with vancomycin against wild-type E. coli. *Antimicrob Agents Chemother* 59, 276–281 10.1128/AAC.03502-14 25348521 PMC4291362

[73] Brynildsen M. P., Winkler J. A., Spina C. S., MacDonald I. C., and Collins J. J. (2013) Potentiating antibacterial activity by predictably enhancing endogenous microbial ROS production. *Nature biotechnology* 31, 160–165 10.1038/nbt.2458 23292609 PMC3568245

[74] Neidhardt F. C., Bloch P. L., and Smith D. F. (1974) Culture medium for enterobacteria. *J Bacteriol* 119, 736–747 10.1128/JB.119.3.736-747.1974 4604283 PMC245675

[75] Sarubbi F. A. Jr., and Hull J. H. (1978) Amikacin serum concentrations: prediction of levels and dosage guidelines. *Ann Intern Med* 89, 612–618 10.7326/0003-4819-89-5-612 717929

[76] Contreras A. M., Gamba G., Cortes J., Santiago Y., Nares F., Jimenez-Sanchez G., et al (1989) Serial trough and peak amikacin levels in plasma as predictors of nephrotoxicity. *Antimicrob Agents Chemother* 33, 973–976 10.1128/aac.33.6.973 2764548 PMC284268

[77] O'Toole G. A. (2011) Microtiter dish biofilm formation assay. *Journal of visualized experiments*: *JoVE* 10.3791/2437 PMC3182663 21307833

[78] Schmitz C., Hilpert J., Jacobsen C., Boensch C., Christensen E. I., Luft F. C., et al (2002) Megalin deficiency offers protection from renal aminoglycoside accumulation. *The Journal of biological chemistry* 277, 618–622 10.1074/jbc.M109959200 11700326

[79] Amidzadeh Z., Behbahani A. B., Erfani N., Sharifzadeh S., Ranjbaran R., Moezi L., et al (2014) Assessment of different permeabilization methods of minimizing damage to the adherent cells for detection of intracellular RNA by flow cytometry. *Avicenna journal of medical biotechnology* 6, 38–46 24523954 PMC3895578

[80] Frye J. G., and Jackson C. R. (2013) Genetic mechanisms of antimicrobial resistance identified in Salmonella enterica, Escherichia coli, and Enteroccocus spp. isolated from U.S. food animals. *Frontiers in microbiology* 4, 135 10.3389/fmicb.2013.00135 23734150 PMC3661942

