# Supplementary material for: Potentiating aminoglycoside antibiotics to reduce their toxic side effects
Source: PLoS One. 2020 Sep 2;15(9):e0237948. doi: 10.1371/journal.pone.0237948 (PMC7467299; doi:10.1371/journal.pone.0237948)
Supplement: S1 File — (PDF) [file pone.0237948.s001.pdf]

Supporting Information for:

Potentiating Aminoglycoside Antibiotics to Reduce Their Toxic Side Effects

Christopher R. Rosenberg,[°,•] Xin Fang,[1,2,•] and Kyle R. Allison[1,2,3,•,*]

1. *Department of Biomedical Engineering, Emory University and Georgia Institute of Technology, Atlanta, GA, USA*
2. *Division of Infectious Diseases, Emory University School of Medicine, Atlanta, GA, USA*
3. *Emory Antibiotic Resistance Center, Atlanta, GA, USA*

° Current affiliation, *Renaissance School of Medicine at Stony Brook University, Stony Brook, NY, USA*

• Previous affiliation *Department of Systems Biology, Columbia University Irving Medical Center, New York, NY, USA*

*Corresponding author: Kyle R. Allison; email: kyle.r.allison@emory.edu; phone: (404) 727-6974

This PDF file includes:

Supporting Figures

Supporting Figures

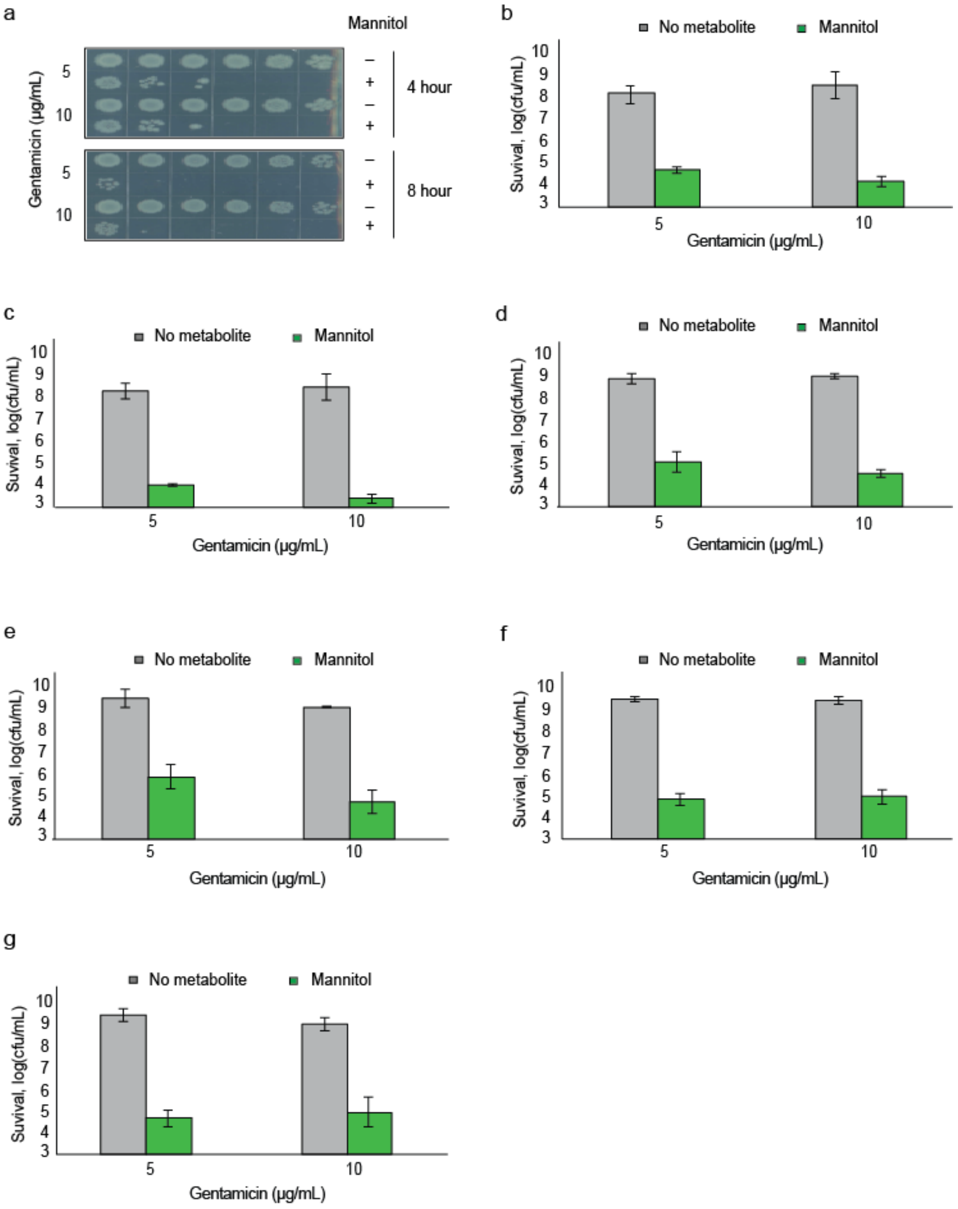

Figure S1. Extended gentamicin treatment nearly sterilizes dormant *E. coli*, but has no additional effect on *S. typhimurium* or *K. pneumoniae*. (a) Image of stationary phase *E. coli* cells following 4- or 8-hour treatment with varying concentrations of gentamicin plus mannitol. **(b)** Survival of stationary phase *E. coli* cells following 4-hour treatment with varying concentrations of gentamicin plus mannitol. **(c)** Survival of stationary

phase *E. coli* cells following 8-hour treatment with varying concentrations of gentamicin plus mannitol. **(d)** Survival of stationary phase *S. typhimurium* cells following 4-hour treatment with varying concentrations of gentamicin plus mannitol. **(e)** Survival of stationary phase *S. tyhimurium* cells following 8-hour treatment with varying concentrations of gentamicin plus mannitol. **(f)** Survival of stationary phase *K. pneumoniae* cells following 4-hour treatment with varying concentrations of gentamicin plus mannitol. **(g)** Survival of stationary phase *K. pneumoniae* cells following 8-hour treatment with varying concentrations of gentamicin plus mannitol. Plots represent mean +/- standard deviation for three or more replicates.

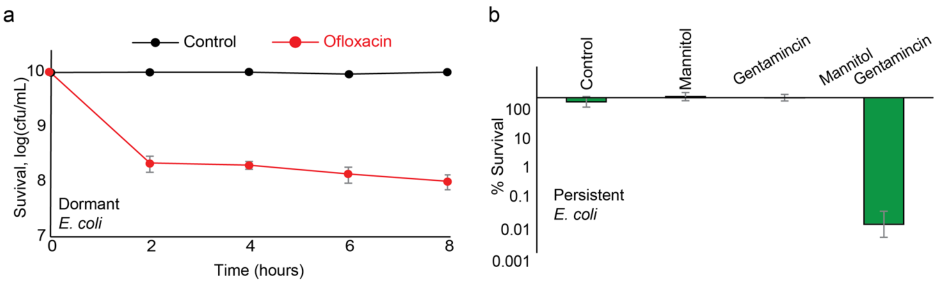

Figure S2. Mannitol potentiates gentamicin against persistent bacteria. **(a)** Survival of stationary-phase *E. coli* cultures after treatment with 5 µg/mL ofloxacin (red line) or no treatment (black line). **(b)** Survival of stationary-phase *E. coli* culture pre-treated with 5 µg/mL ofloxacin for 4 hours, then treated with no metabolite, 10 mM mannitol, 10 µg/mL gentamicin, and mannitol plus gentamicin. Plots represent mean +/- standard deviation for three or more replicates.

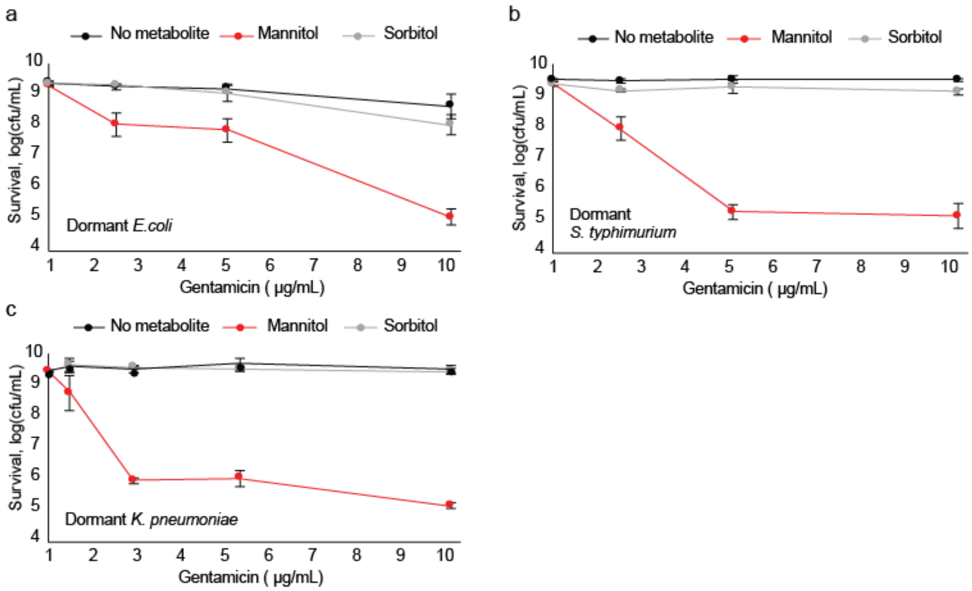

Figure S3. Sorbitol does not reduce gentamicin concentration required for treatment of dormant *E. coli, S. typhimurium, or K. pneumoniae.* **(a)** Survival of stationary phase *E. coli* cells after 2-hour treatment with varying concentrations of gentamicin and no metabolite (black line), 10 mM sorbitol (grey line), and 10 mM mannitol (red line). **(b)** Survival of stationary phase *S. typhimurium* cells after 2-hour treatment with varying concentrations of gentamicin plus metabolite. **(c)** Survival of stationary phase *K. pneumoniae* cells after 2-hour treatment with varying concentrations of gentamicin plus metabolite. Plots represent mean +/- standard deviation for three or more replicates.

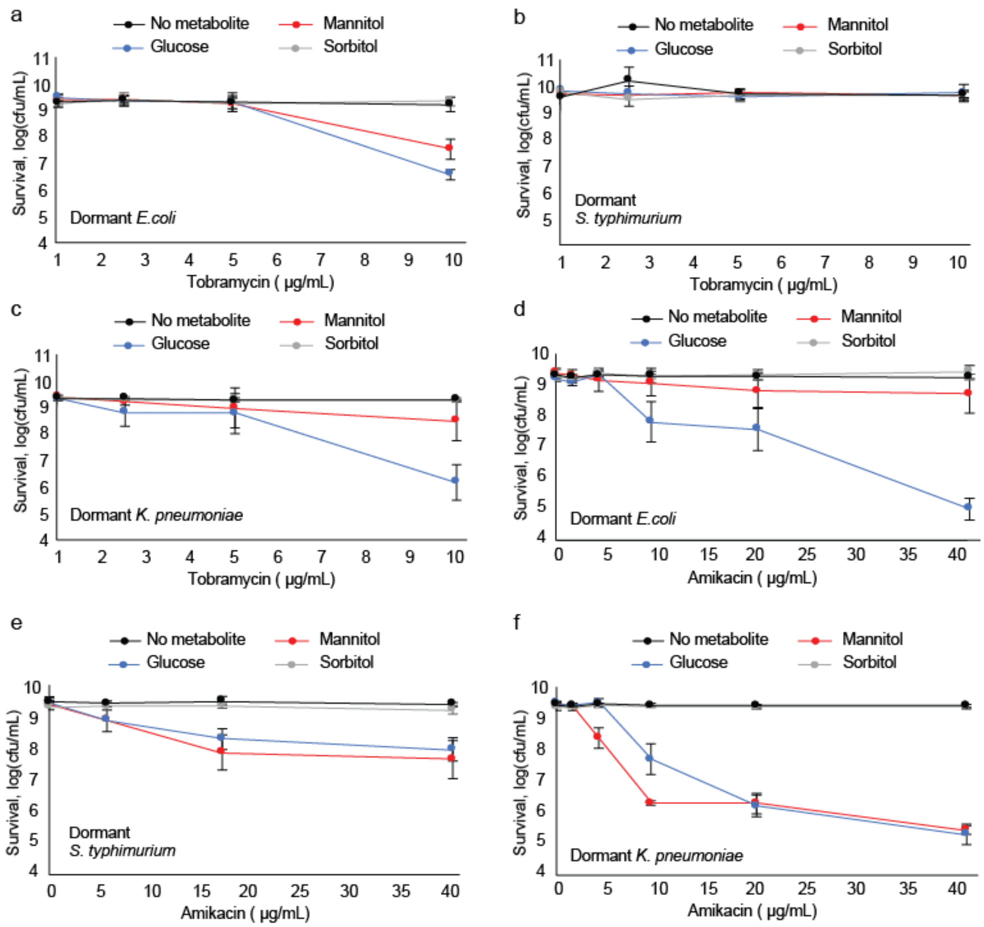

Figure S4. Metabolite potentiation applies broadly to the aminoglycoside class. **(a)** Survival of stationary phase *E. coli* cells after 2-hour treatment with varying concentrations of tobramycin and no metabolite (black line), 10 mM glucose (blue line), 10 mM sorbitol (grey line), and 10 mM mannitol (red line). **(b)** Survival of stationary phase *S. typhimurium* cells after 2-hour treatment with varying concentrations of tobramycin plus metabolite. **(c)** Survival of stationary phase *K. pneumoniae* cells after 2-hour treatment with varying concentrations of tobramycin plus metabolite. **(d)** Survival of stationary phase *E. coli* cells after 2-hour treatment with varying concentrations of amikacin with or without metabolite. **(e)** Survival of stationary phase *S. typhimurium* cells after 2-hour treatment with varying concentrations of amikacin plus metabolite. **(f)** Survival of stationary phase *K. pneumoniae* cells after 2-hour treatment with

varying concentrations of amikacin plus metabolite. Plots represent mean +/- standard deviation for three or more replicates.

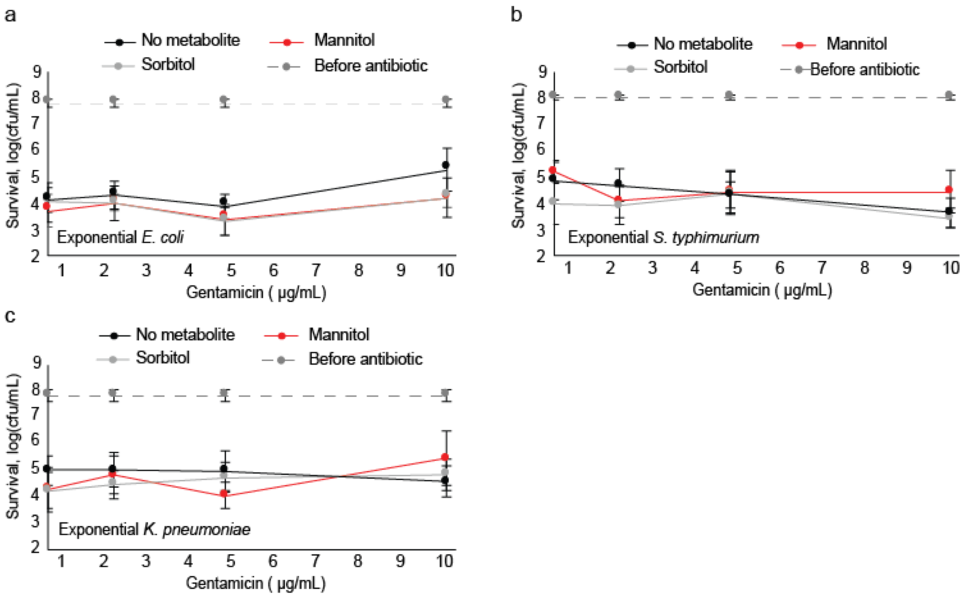

Figure S5. Metabolite supplementation does not promote growth or tolerance in rapidly growing bacteria. (a) Survival of exponential phase *E. coli* cells after 2-hour treatment with varying concentrations of gentamicin and no metabolite (black line), 10 mM mannitol (red line), or 10 mM sorbitol (grey line). Initial cell viability before treatment is indicated (grey dashed line). **(b)** Survival of exponential phase *S. typhimurium* cells after 2-hour treatment with varying concentrations of gentamicin plus metabolite. **(c)** Survival of exponential phase *K. pneumoniae* cells after 2-hour treatment with varying concentrations of gentamicin plus metabolite. Plots represent mean +/- standard deviation for three or more replicates.

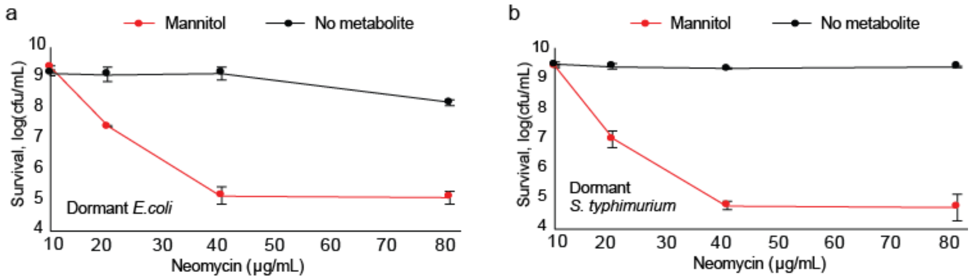

Figure S6. Mannitol potentiation treats antibiotic-tolerant Gram-negative bacteria at significantly reduced neomycin concentrations. (a) Survival of stationary phase *E. coli* cells after 2-hour treatment with varying concentrations of gentamicin and 10 mM mannitol (red line) or no metabolite (black line). **(b)** Survival of stationary phase *S. typhimurium* cells after 2-hour treatment with varying concentrations of gentamicin plus mannitol. Plots represent mean +/- standard deviation for three or more replicates.

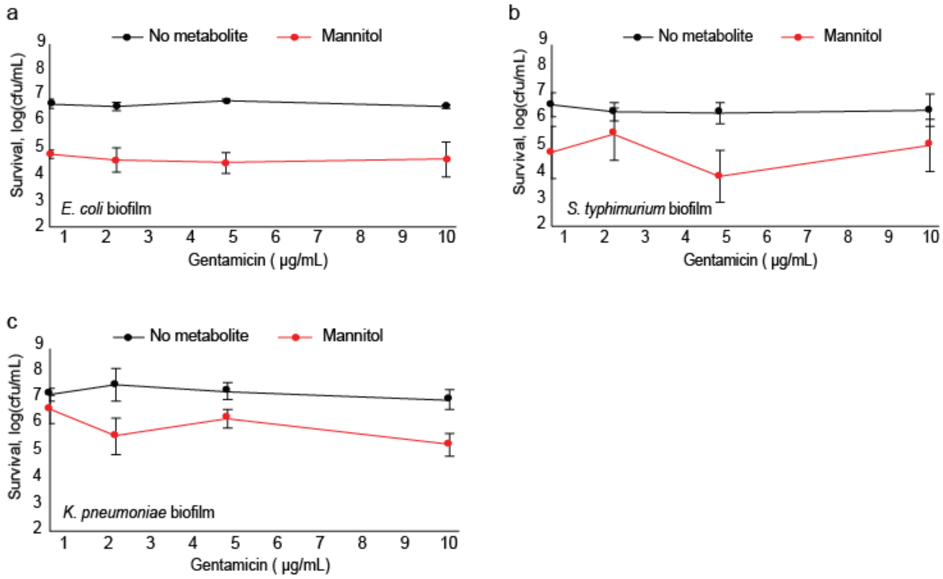

Figure S7. Mannitol potentiation treats Gram-negative biofilms at significantly reduced gentamicin concentrations. **(a)** Survival of *E. coli* biofilm cells after 2-hour treatment with varying concentrations of gentamicin and 10 mM mannitol (red line) or no metabolite (black line). **(b)** Survival of *S. typhimurium* biofilm cells after 2-hour treatment with varying concentrations of gentamicin plus mannitol. **(c)** Survival of *K. pneumoniae* biofilm cells after 2-hour treatment with varying concentrations of gentamicin plus mannitol. Plots represent mean +/- standard deviation for three or more replicates.

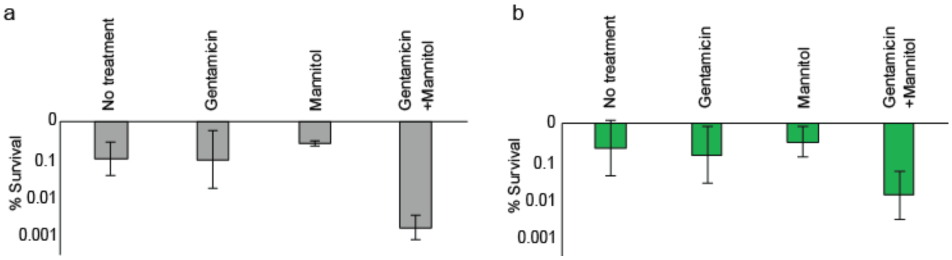

Figure S8. Liberated biofilm cells do not proliferate following aminoglycoside-metabolite treatment. **(a)** Survival of *E. coli* biofilm supernatant cells after 2-hour treatment with gentamicin (5 µg/mL) plus mannitol (10 mM). **(b)** Survival of *K. pneumoniae* biofilm supernatant cells after 2-hour treatment with gentamicin plus mannitol. Plots represent mean +/- standard deviation for three or more replicates.
